# Supplementary material for: Gap-enhanced Raman tags for physically unclonable anticounterfeiting labels
Source: Nat Commun. 2020 Jan 24;11:516. doi: 10.1038/s41467-019-14070-9 (PMC6981139; doi:10.1038/s41467-019-14070-9)
Supplement: Supplementary file 1 — Supplementary information [file 41467_2019_14070_MOESM1_ESM.pdf]

Supplementary Information

Gap-enhanced Raman tags for physically unclonable anticounterfeiting labels

Yuqing Gu et al.

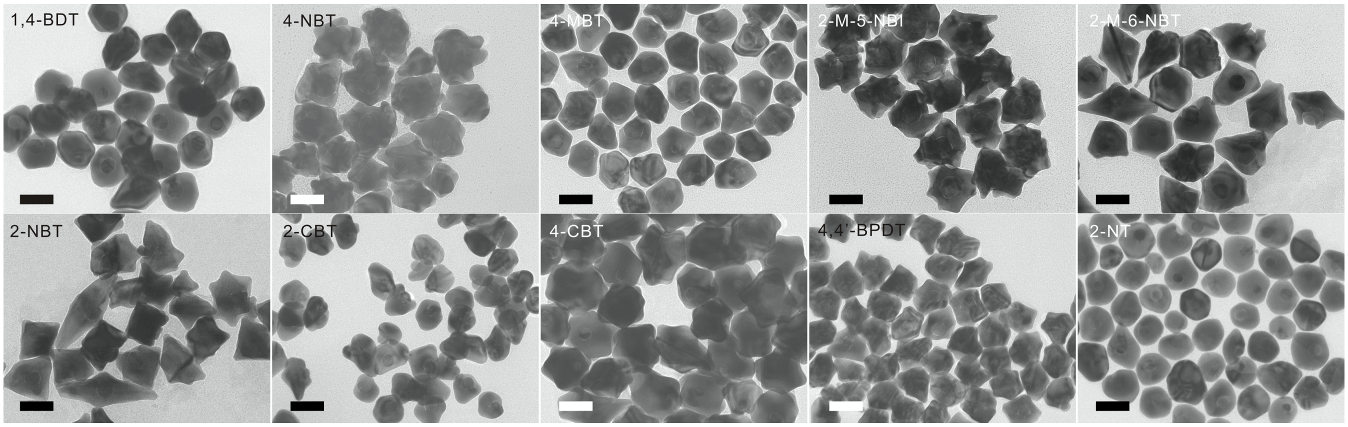

Supplementary Figure 1. TEM images of GERTs with different embedded reporter molecules. All scale bars are 50 nm.

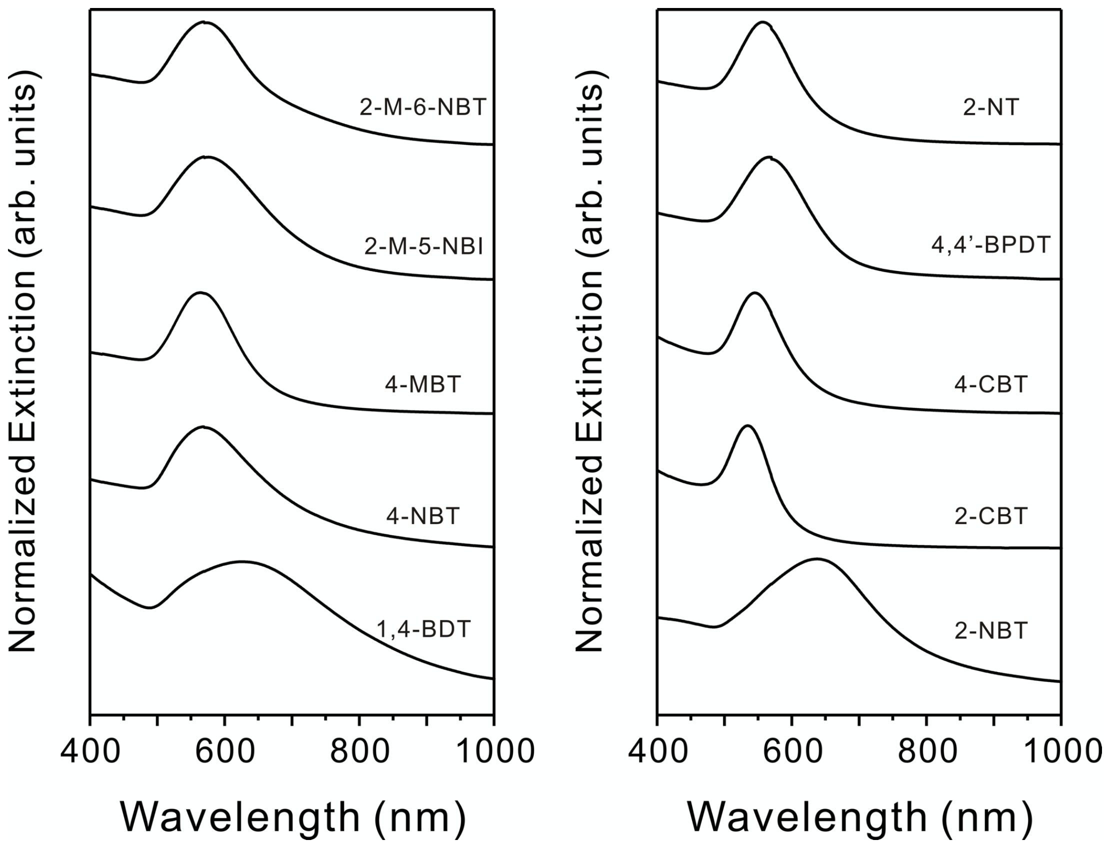

Supplementary Figure 2. Extinction spectra of GERTs with different embedded reporter molecules.

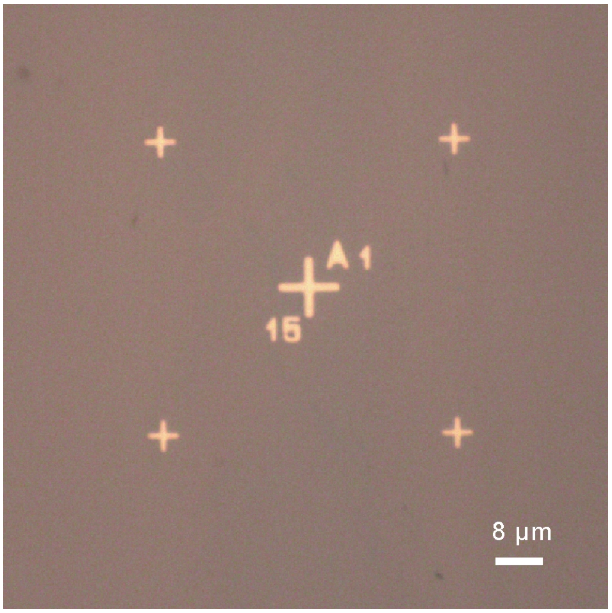

Supplementary Figure 3. The silica substrate with metallic marks for fabrication of PUF labels.

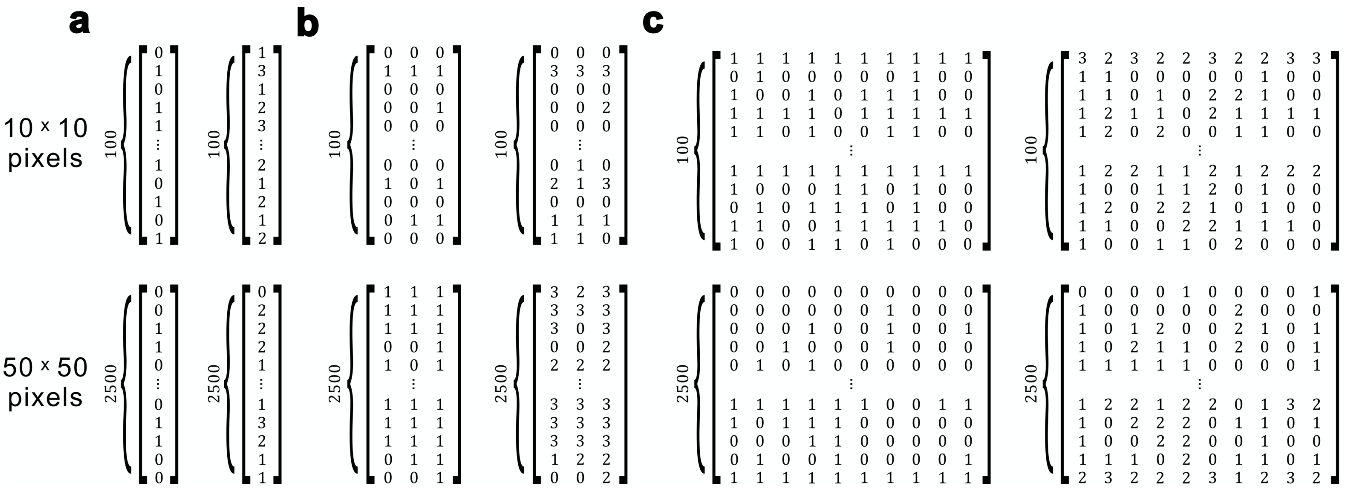

Supplementary Figure 4. Matrices for digitization of binary (left column) and quaternary (right column) encoding of Raman mappings with a resolution of 10 × 10 (top column) and 50 × 50 pixels (bottom column) for (**a**) one-type GERT, (**b**) three-type GERT, and (**c**) ten-type GERT PUF labels, respectively.

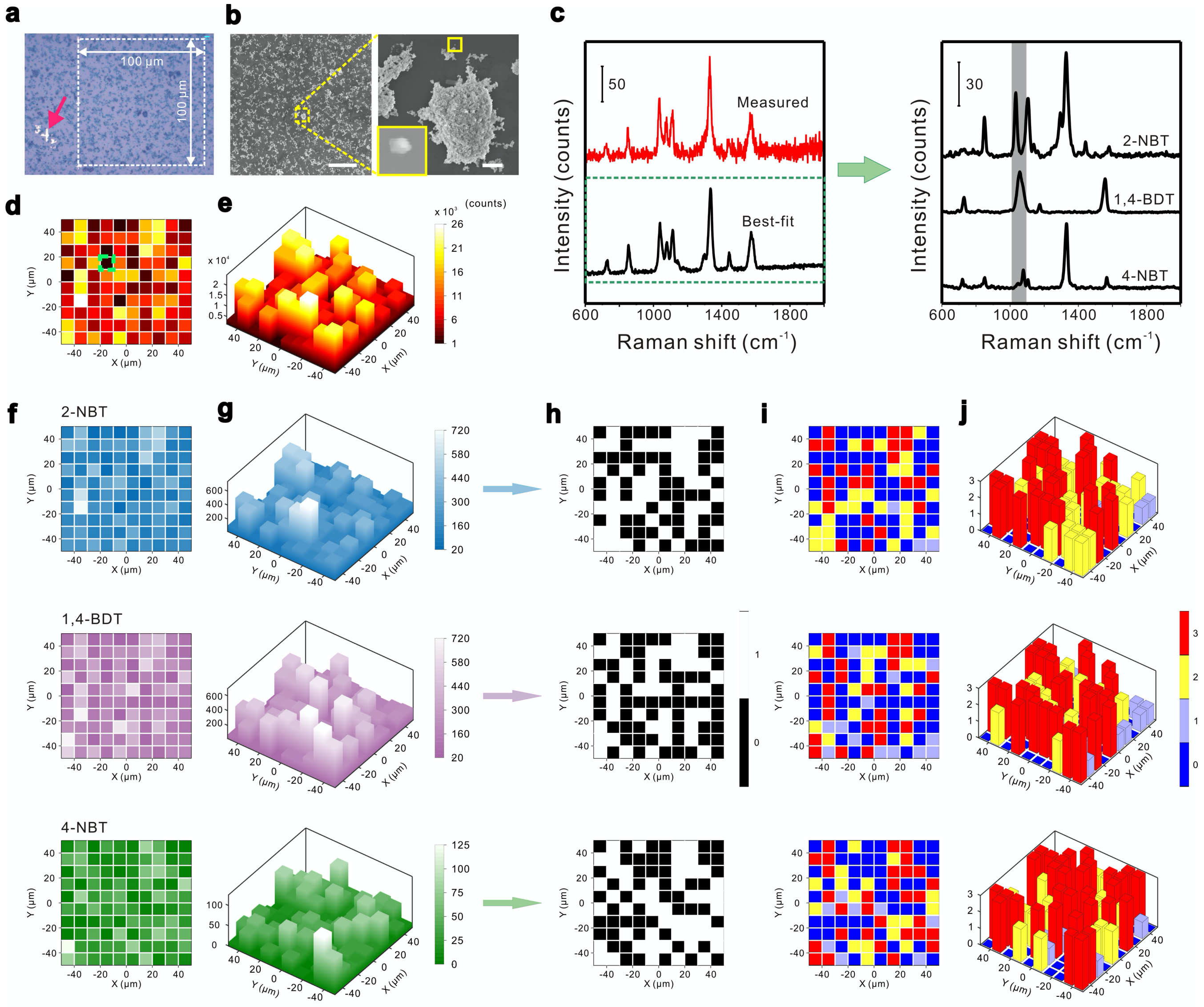

Supplementary Figure 5. Fabrication and digitization of a PUF label composed of three types of GERTs. (**a**) A bright-field image and (**b**) the scanning electron microscopy image (corresponding to the dashed square area in **a**) of a PUF label fabricated using three types of GERTs (1,4-BDT GERTs, 4-NBT GERTs, and 2-NBT GERTs) in an area of 100 × 100 μm^2 on a SiO_2 substrate. Scale bars are 20 (left) and 1 μm (right). (**c**) Spectral demultiplexing to quantify the abundance of three types of GERTS. The best-fit spectrum (black) is obtained by fitting the measured Raman spectrum (blue) with pure reference Raman spectra of 2-NBT GERTs, 1,4-BDT GERTs, and 4-NBT GERTs using the non-negative least squares (NNLS) method. (**d**) 2D and (**e**) 3D plot for the readout of the PUF label by the Raman mapping (with a resolution of 10 × 10 pixels) of (i) multiplexed measured Raman signals using the integrated areas of Raman bands from 1018 to 1090 cm^{-1} (as indicated in the grey area in panel (**c**). (**f**) 2D and (**g**) 3D plot for the readout of the PUF label by the Raman mapping of demultiplexed Raman signals of 2-NBT GERTs, 1,4-BDT GERTs, and 4-NBT GERTs. (**h**, **i**) 2D and (**j**) 3D plot of the digitization of the corresponding demultiplexed Raman signals using (**h**) binary encoding and (**i**, **j**) quaternary encoding of Raman intensity levels at each pixel.

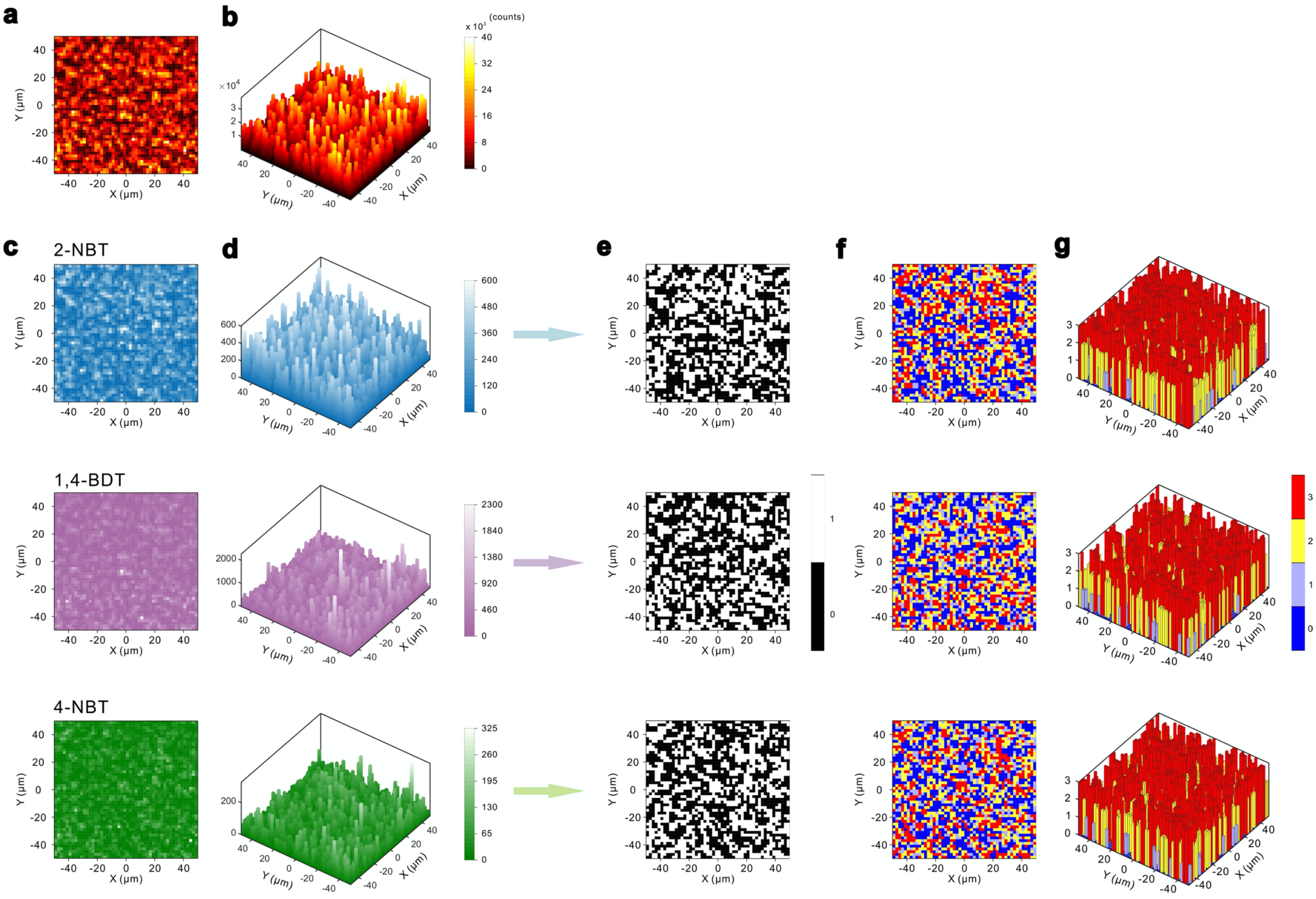

Supplementary Figure 6. (**a**) 2D and (**b**) 3D plot for the readout of the same PUF label in Figure S5 by the Raman mapping (with a resolution of 50 × 50 pixels) of multiplexed measured Raman signals. (**c**) 2D and (**d**) 3D plot for the readout of the PUF label by the Raman mapping of demultiplexed Raman signals of 2-NBT GERTs, 1,4-BDT GERTs, and 4-NBT GERTs. (**e**, **f**) 2D and (**g**) 3D plot for the digitization of the corresponding demultiplexed Raman signals for (**e**) binary encoding and (**f**, **g**) quaternary encoding of Raman intensity levels at each pixel.

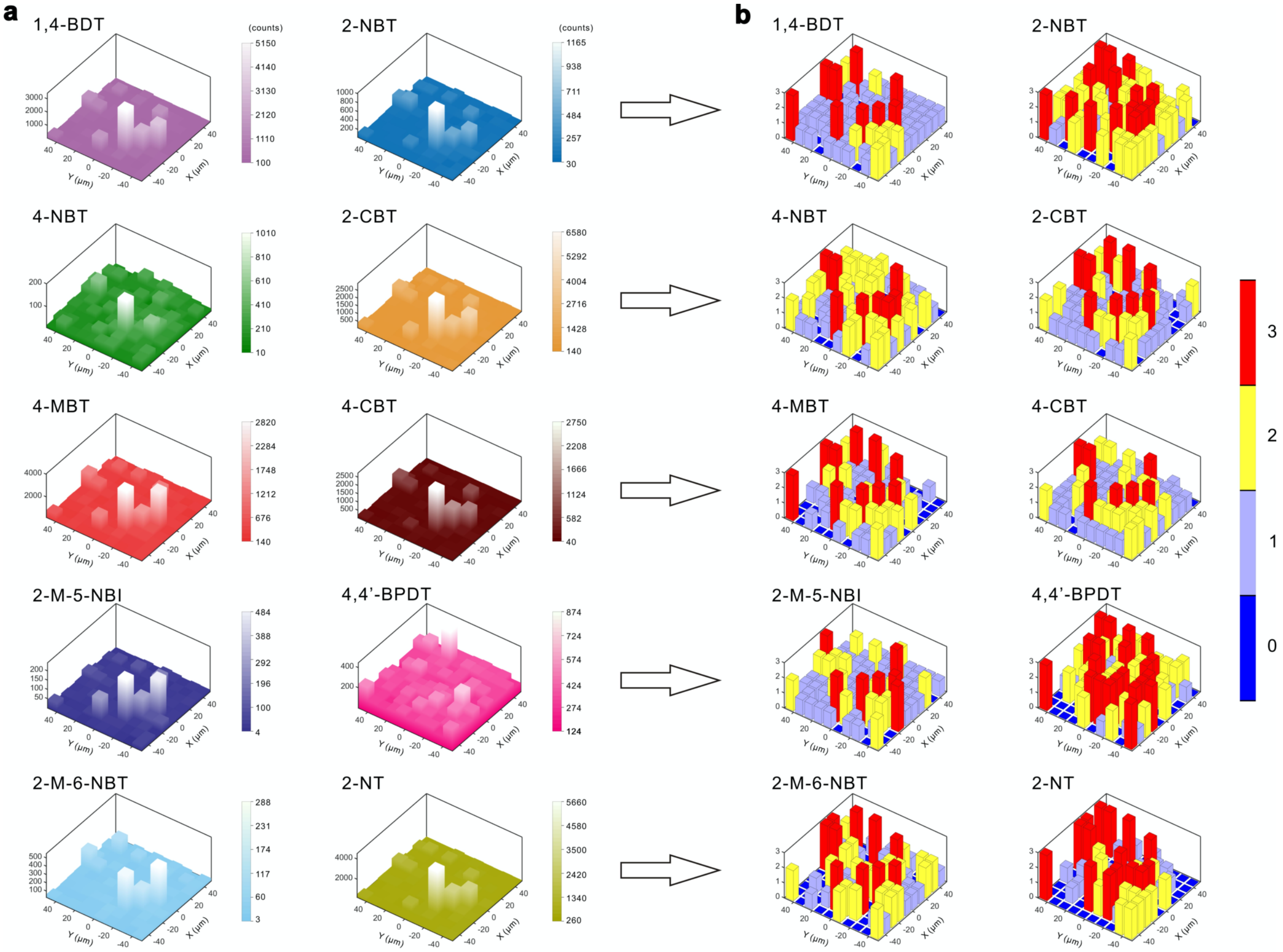

Supplementary Figure 7. (**a**) 3D plot for the readout of the PUF label composed of ten types of GERTs by the Raman mapping (with a resolution of 10 × 10 pixels) and (**b**) 3D plot for the digitization of the corresponding quaternary encoding of Raman intensity levels at each pixel based on demultiplexed Raman signals of ten different GERTs.

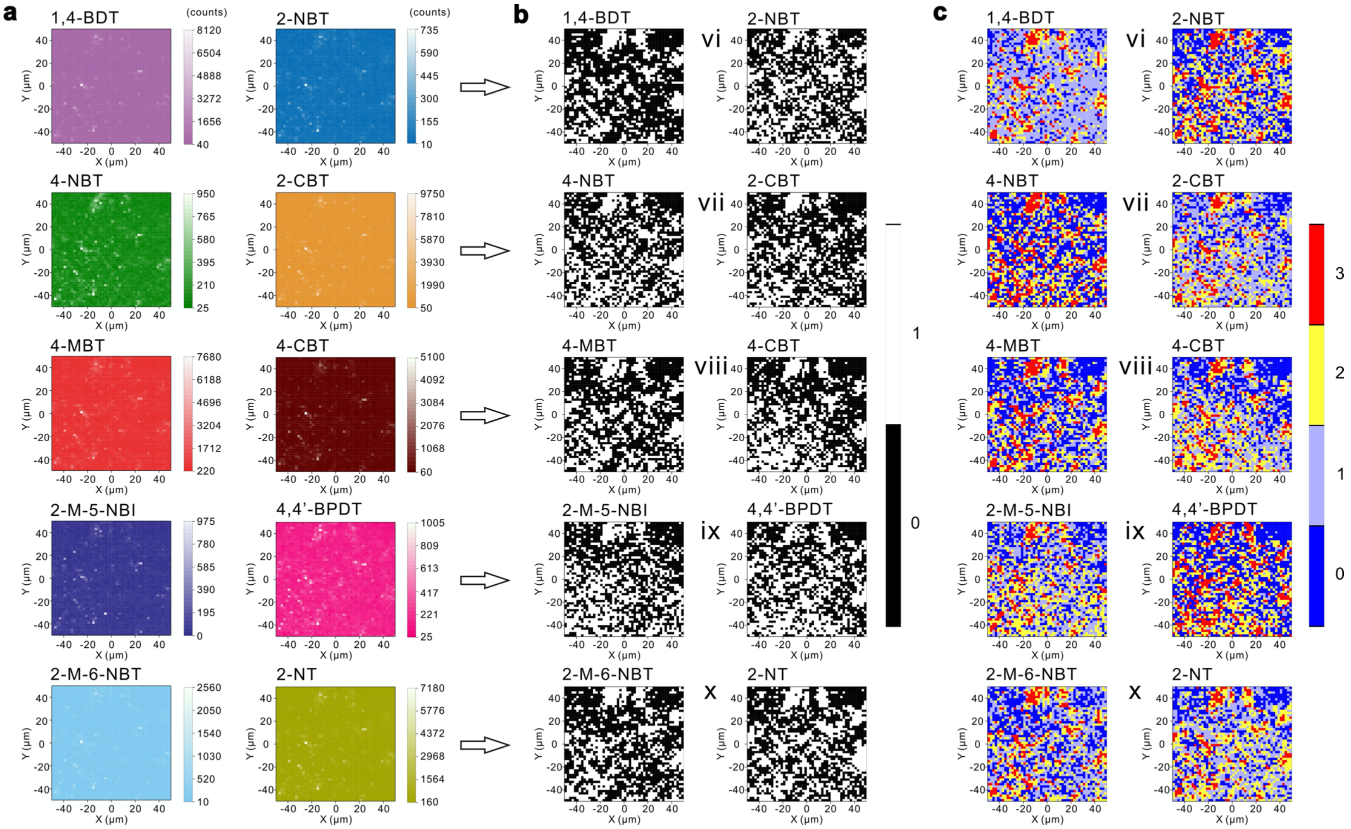

Supplementary Figure 8. Digitization of a PUF label composed of ten types of GERTs. (**a**) 2D plot for the readout of the PUF label by the Raman mapping (with a resolution of 50 × 50 pixels) and 2D plot for the digitization of the corresponding (**b**) binary encoding and (**c**) quaternary encoding of Raman intensity levels at each pixel based on demultiplexed Raman signals of ten different GERTs.

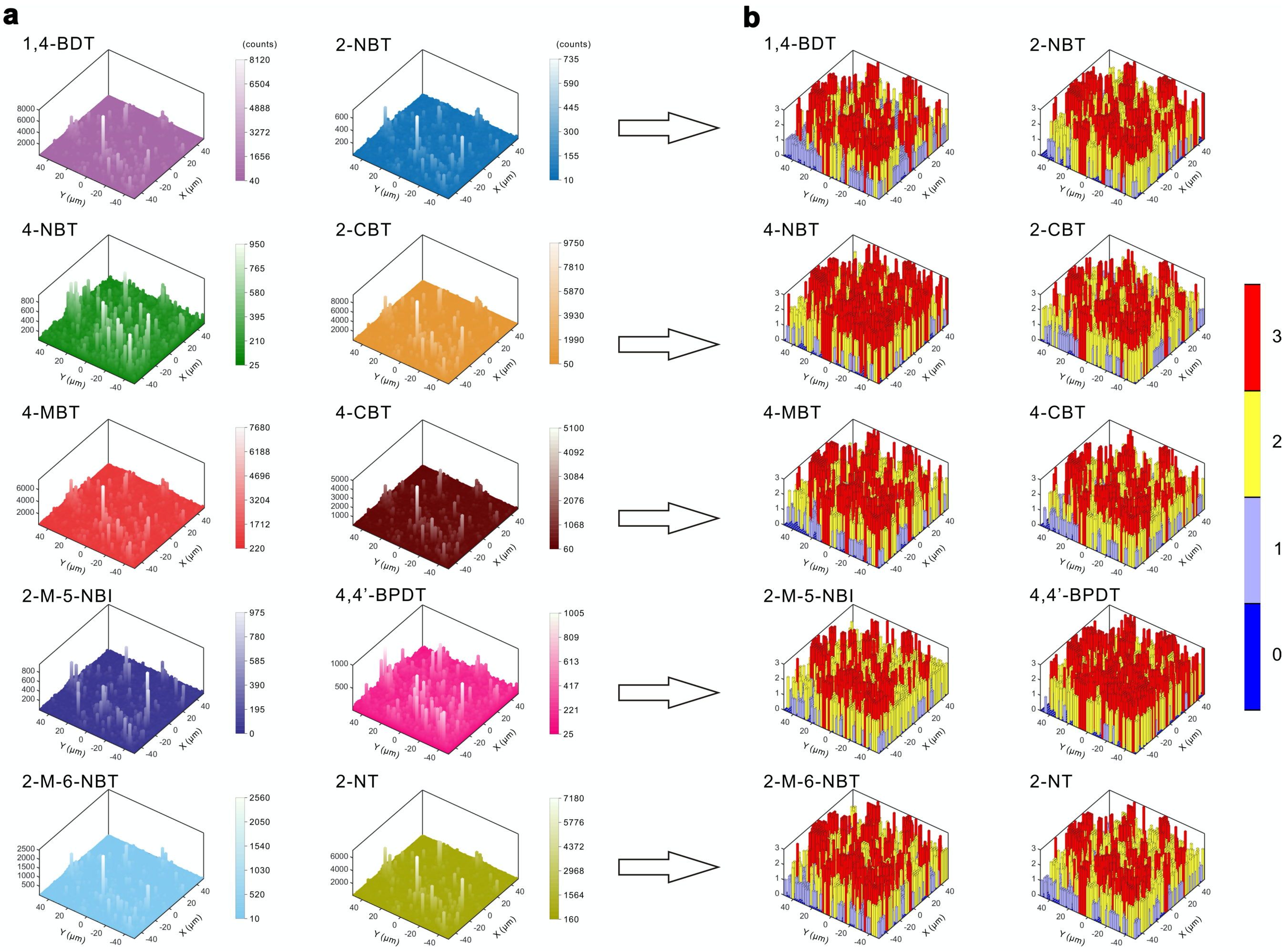

Supplementary Figure 9. (**a**) 3D plot for the readout of the PUF label composed of ten types of GERTs by the Raman mapping (with a resolution of 50 × 50 pixels) and (**b**) 3D plot for the digitization of the corresponding quaternary encoding of Raman intensity levels at each pixel based on demultiplexed Raman signals of ten different GERTs.

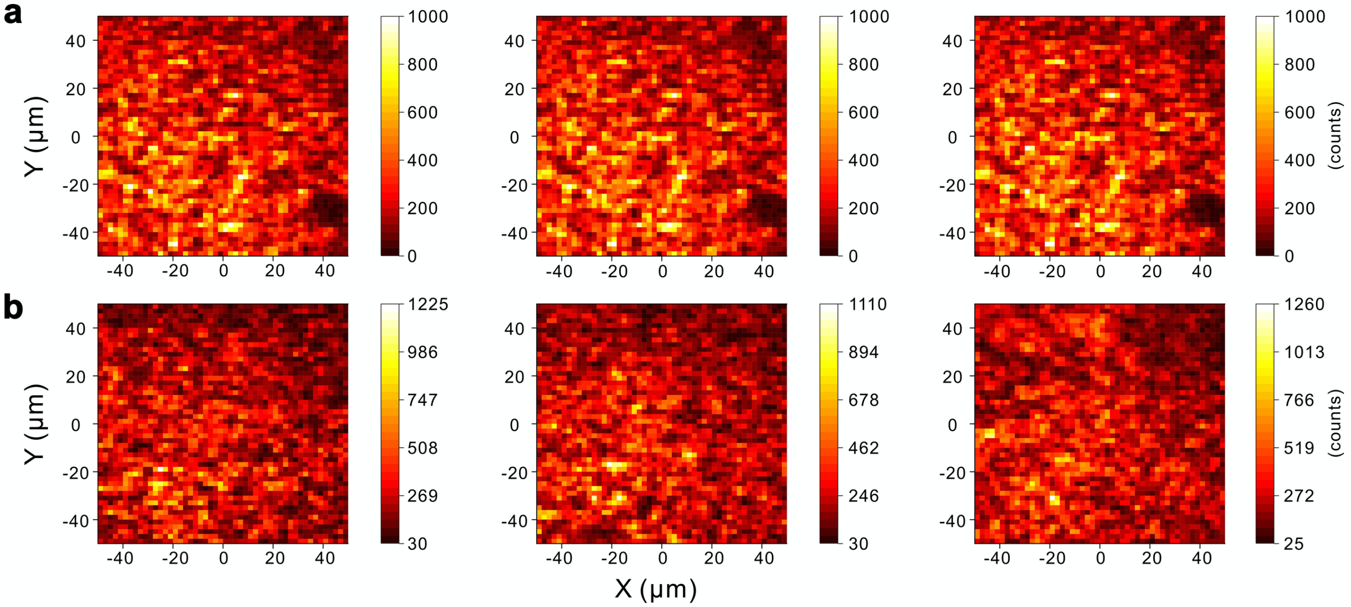

Supplementary Figure 10. (**a**) Readout of the PUF label presented in Figure 5a and b with a resolution of 50 × 50 pixels for the first (left), second (middle), and third (right) measurement. (**b**) Readout of additional three labels numbered 2 (left), 3 (middle), and 4 (right) presented in Figure 5c and d with a resolution of 50 × 50 pixels.

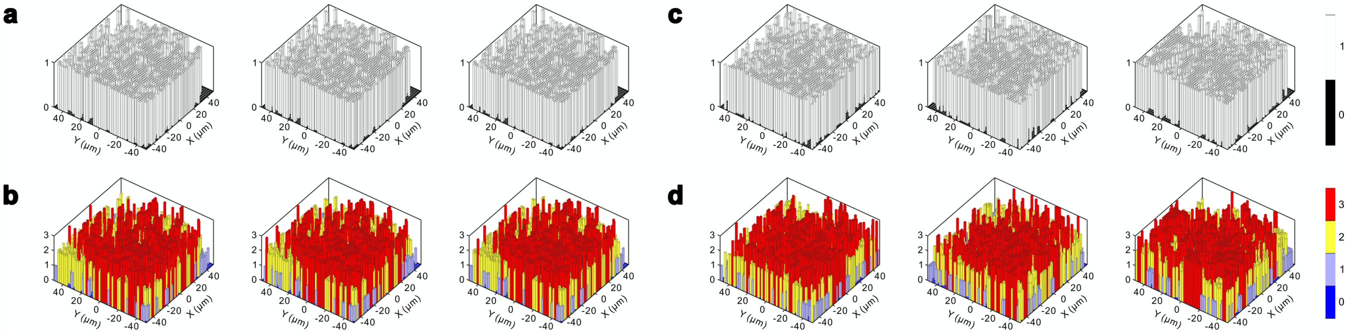

Supplementary Figure 11. 3D plot for the digitization of the PUF label presented in Figure 5a and b with a resolution of 50 × 50 pixels in (**a**) binary and (**b**) quaternary encoding of Raman intensity levels at each pixel for the first (left), second (middle), and third (right) measurement. 3D plot for the digitization of additional three PUF labels numbered 2 (left), 3 (middle), and 4 (right) presented in Figure 5c and d with a resolution of 50 × 50 pixels in (**c**) binary and (**d**) quaternary encoding of Raman intensity levels at each pixel.

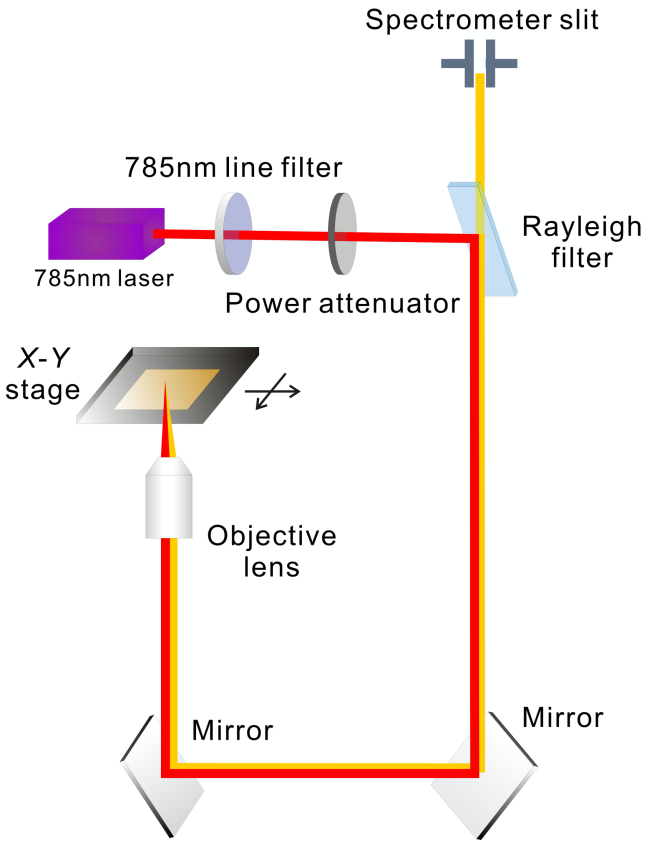

Supplementary Figure 12. Schematic of configurations of Raman system used for conventional scanning.

Supplementary Table 1 | Main peaks and the corresponding vibrational assignments of the GERTs.

Raman reporter	Raman shift (cm^{-1})	Assignment
1,4-BDT	1058	phenyl-ring breathing mode (ν_1)[1]
	1558	phenyl-ring stretching motion (ν_{8a})[1]
4-NBT	1078	CS stretching[2]
	1331	symmetric stretching of the nitro group [ν_s (NO_2)][3]
	1575	CC stretching of the phenyl-ring[3]
4-MBT	1077	ν_1[4]
2-M-5-NBI	817	phenyl-ring bending mode[5]
	1283	CNH in-plane bending[5]
2-M-6-NBT	1123	CH in-plane bending[6]
	1300	CS stretching[6]

2-NBT	1033	CC stretching + SH bending[7]
	1104	CS stretching[7]
	1328	ν_s (NO_2)[7]
2-CBT	1036	phenyl-ring def in-plane mode[8]
	1078	ν_a (CC) stretching[9]
	1104	ν_a (CS) stretching[9]
	1566	ν (CC) stretching[9]
4-CBT	1060	ν_{12}[10]
	1082	ν_1[10]
	1567	ν_{8a}[10]
4,4'-BPDT	1080	CS stretching[11]
	1585	aromatic tangential phenyl-ring stretching (C=C)[11]
2-NT	1066	CH bending[12]
	1328	ν_{8a}[13]
	1379	ν_{8a}[13]

Supplementary Table 2 | The similarity index *I* between PUF labels in Fig. 5.

	**Binary (%)**	**Quaternary (%)**
$I_{11'\text{-}1}$	94.76	85.48
$I_{11'\text{-}2}$	94.12	84.36
$I_{11'\text{-}3}$	94.64	85.48
I_{12}	57.64	30.44
I_{13}	58.92	31.32
I_{14}	56.48	30.24

$I_{11'\text{-}1}$, $I_{11'\text{-}2}$ and $I_{11'\text{-}3}$ represent the similarity indexes between the first and the second, the first and the third, the second and the third measurement of the same PUF label displayed in Fig. 5a and 5b, respectively. I_{12}, I_{13} and I_{14} represent the similarity indexes between the first measurement of the label displayed in Fig. 5a and 5b and additional three labels.

Supplementary References

1. Zhang, Y. et al. Ultraphotostable mesoporous silica-coated gap-enhanced Raman tags (GERTs) for high-speed bioimaging. *ACS applied materials & interfaces* **9**, 3995-4005 (2017).
2. Kuo, S.-C. et al. Enhancement of surface enhanced Raman scattering activity of Au nanoparticles through the mesostructured metallic nanoparticle arrays. *APL Materials* **2**, 113310 (2014).
3. Kim, K., Choi, J.-Y. & Shin, K.S. Surface-enhanced Raman scattering of 4-nitrobenzenethiol and 4-aminobenzenethiol on silver in icy environments at liquid nitrogen temperature. *The Journal of Physical Chemistry C* **118**, 11397-11403 (2014).
4. Fan, W. et al. Graphene oxide and shape-controlled silver nanoparticle hybrids for ultrasensitive single-particle surface-enhanced Raman scattering (SERS) sensing. *Nanoscale* **6**, 4843-4851 (2014).
5. Wu, W. et al. Low-cost, disposable, flexible and highly reproducible screen printed SERS substrates for the detection of various chemicals. *Scientific reports* **5**, 10208 (2015).
6. Mohan, S., Prabakaran, A. & Payami, F. Raman and infrared spectra of 2 - amino - 6 - nitrobenzothiazole, normal coordinate analysis and vibrational assignments. *Journal of Raman spectroscopy* **20**, 455-459 (1989).
7. Teguh, J.S., Liu, F., Xing, B. & Yeow, E.K. Surface - Enhanced Raman Scattering (SERS) of Nitrothiophenol Isomers Chemisorbed on TiO2. *Chemistry–An Asian Journal* **7**, 975-981 (2012).
8. Bu, Y., Park, S.J. & Lee, S.-W. Diamine-linked array of metal (Au, Ag) nanoparticles on glass substrates for reliable surface-enhanced Raman scattering (SERS) measurements. *Current Applied Physics* **14**, 784-789 (2014).
9. Bu, Y. & Lee, S. Optimizing surface-enhanced RAMAN scattering-active Au nanostructures coated on indium-doped tin oxide glass by combining chemical assembly and electrodeposition methods. *Japanese Journal of Applied Physics* **52**, 10MD02 (2013).
10. Szafranski, C.A., Tanner, W., Laibinis, P.E. & Garrell, R.L. Surface-enhanced Raman spectroscopy of halogenated aromatic thiols on gold electrodes. *Langmuir* **14**, 3580-3589 (1998).
11. Cui, L. et al. In situ gap-mode raman spectroscopy on single-crystal Au (100) electrodes: Tuning the torsion angle of 4, 4′ -biphenyldithiols by an electrochemical gate field. *Journal of the American Chemical Society* **133**, 7332-7335 (2011).
12. Harun, N.A., Horrocks, B.R. & Fulton, D.A. Enhanced Raman and luminescence spectra from co-encapsulated silicon quantum dots and Au–Ag nanoalloys. *Chemical Communications* **50**, 12389-12391 (2014).
13. Alvarez-Puebla, R., Dos Santos Jr, D. & Aroca, R. Surface-enhanced Raman scattering for ultrasensitive chemical analysis of 1 and 2-naphthalenethiols. *Analyst* **129**, 1251-1256 (2004).
